# RailEnV-PASMVS: A perfectly accurate, synthetic, path-traced dataset featuring a virtual railway environment for multi-view stereopsis training and reconstruction applications

**DOI:** 10.1016/j.dib.2021.107411

**Published:** 2021-09-23

**Authors:** André Broekman, Petrus Johannes Gräbe

**Affiliations:** a Department of Civil Engineering, Engineering 4.0, University of Pretoria, Lynnwood Road, Hatfield, Pretoria 0002, South Africa; b Civil and Environmental Engineering, University of Southampton

**Keywords:** Multi-view stereopsis, Railway engineering, Semantic segmentation, Synthetic data, Ground truth depth maps, Geolocation, ECEF, Blender

## Abstract

A Perfectly Accurate, Synthetic dataset featuring a virtual railway EnVironment for Multi-View Stereopsis (RailEnV-PASMVS) is presented, consisting of 40 scenes and 79,800 renderings together with ground truth depth maps, extrinsic and intrinsic camera parameters, pseudo-geolocation metadata and binary segmentation masks of all the track components. Every scene is rendered from a set of 3 cameras, each positioned relative to the track for optimal 3D reconstruction of the rail profile. The set of cameras is translated across the 100 m length of tangent (straight) track to yield a total of 1995 camera views. Photorealistic lighting of each of the 40 scenes is achieved with the implementation of high-definition, high dynamic range (HDR) environmental textures. Additional variation is introduced in the form of camera focal lengths, camera location and rotation parameters and shader modifications for materials. Representative track geometry provides random and unique vertical alignment data for the rail profile for every scene. This primary, synthetic dataset is augmented by a smaller photograph collection consisting of 320 annotated photographs for improved semantic segmentation performance. The combination of diffuse and specular properties increases the ambiguity and complexity of the data distribution. RailEnV-PASMVS represents an application specific dataset for railway engineering, against the backdrop of existing datasets available in the field of computer vision, providing the precision required for novel research applications in the field of transportation engineering. The novelty of the RailEnV-PASMVS dataset is demonstrated with two use cases, resolving shortcomings of the existing PASMVS dataset.

## Graphical abstract

**Figure:**
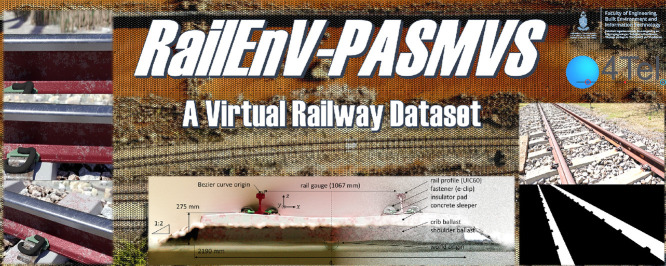

## Specifications Table

**Table:** 

Subject	Computer Vision and Pattern Recognition
Specific subject area	Multi-view stereopsis and 3D reconstruction from images
Type of data	Image Depth maps CSV
How data were acquired	A virtual railway environment was reconstructed within Blender using a mixture of modelled and scanned railway components, each with realistic material shaders, based on the methodology implemented by PASMVS [1]. The path-tracing rendering engine (Cycles) was used to render the colour images, binary segmentation masks and depth maps for all camera perspectives. A variety of background environmental textures, randomised camera parameters (location and rotation), material variation and unique vertical alignment geometry add the requisite variation. The rail components (rail profile, e-clip fastener, insulator pad concrete sleeper, ballast and ground surface) were assigned unique identifiers to generate individual binary segmentation masks. The ground truth depth map was acquired from the Z-buffer (distance between the intersecting geometry and camera for every pixel comprising the imaging sensor) associated with the camera for each rendering pass. Extrinsic and intrinsic camera information are exported as an aggregated CSV (comma-separated value) file for every scene. Equivalent geolocation information is embedded in the image's EXIF (exchangeable image file format) properties during the post-processing phase. The photographic subset was acquired from three locations installations using digital cameras (hand operated and aerial perspectives); the corresponding binary segmentation maps were hand annotated for the rail profile.
Data format	Raw Annotated
Parameters for data collection	Variation of the vertical alignment of the track, in combination with translating the camera along the length of the railway track, served as the primary variables. Camera locations and rotations, camera focal length and environmental background textures increase the data distribution and the associated variability of the dataset to improve the robustness of reconstruction algorithms and pipelines. The photographic subset is a combination of diffuse and specular railway properties typically encountered in railway environments, representing various illumination conditions.
Description of data collection	A virtual railway environment was created in Blender for the purpose of generating (rendering) path-traces images, binary segmentation masks and ground truth depth maps for different vertical alignment geometry associated with the rail profile. 40 scenes were rendered using a combination of 1995 cameras translated over a section of tangent track measuring 100 m in length, yielding a total of 79,800 synthetic samples (covering a total of 4 km of track) for the dataset. Every scene is associated with a unique environment texture for photorealistic illumination. The camera parameters are exported to generate the corresponding matrices and equivalent geolocation metadata
	for the rendered samples. The rendered distance maps exported from Blender are post-processed to produce the correct depth maps used for training applications and quantification of accuracy. The photographic dataset is comprised of 320 high-resolution photographs, each with a corresponding binary segmentation map (hand annotated) of the rail profile.
Data source location	Institution: Department of Civil Engineering, Engineering 4.0, University of Pretoria City: Pretoria Country: South Africa
Data accessibility	Repository name: Zenodo Data URL: https://doi.org/10.5281/zenodo.5233840

## Value of the Data

• The dataset enables the development of accurate, sub-millimetre accurate reconstruction pipelines and architectures for specific applications (railway environments) that implement sensitive optical metrology.
• RailEnV-PASMVS [2] can be used for training MVS neural network architectures [3,4] and benchmarking photogrammetric pipelines that are dependent on large, accurate ground truth datasets, in addition to structure-from-data pipelines dependant on accurate geolocation metadata provided that is embedded in the dataset.
• The data structure and file formats are identical to BlendedMVS [5] and PASMVS [1], which are agnostic to state-of-the-art, MVS neural network implementation requirements [3,4].
• RailEnV-PASMVS resolves select shortcomings presented by the PASMVS dataset, specifically highly specular and shadowed regions in addition to providing a larger number of training examples for improved generalisation for varied environmental illumination conditions.

## 1 Data Description

MVS reconstruction pipelines, particularly state-of-the-art developments that are based on neural network implementations, require both a large sample distribution of photorealistic image sequences alongside accurate ground truth depth maps, to learn and generalise effectively [3,4]. Datasets such as Blended MVS illustrate the ease with which datasets can be collected, subject to limitations of photogrammetric reconstruction [5]. PASMVS [1] by comparison presented a flexible, open-source development pipeline to generate equivalent, synthetic datasets that offer the benefit of absolute accuracy for improved precision. Datasets such as IVL-SYNTHSFM-v2 [6] follow a similar development process. The need for physical instrumentation which functions as the primary medium for capturing physical data, such as cameras, unmanned aerial vehicles (UAVs) [5] or laser-based scanning methods [7], are eliminated entirely from the process. Dataset domains tend to reflect applicable research areas of interest, in particular the automotive sector. Compared to other areas of transportation engineering, for example the railway sector, data acquisition for these environments presents a much lower technical and legislative barrier to collect either physical data (for example KITTI [8]) or synthetic data sourced from video game engines [9]. Based on the proven performance and flexible performance presented by Blended MVS [5] and PASMVS [1] respectively, RailEnV-PASMVS [2] was developed for the domain specific application of railway engineering. RailEnV-PASMVS presents improvements over other datasets, directly embedding EXIF (exchangeable image file format) metadata of the images (geolocation and optical properties), in addition to the mixture of both diffuse and specular materials. Existing datasets [5] typically relegate the image properties to separate files with the underlying photogrammetric reconstruction methods not embedding specular information. The development of RailEnV-PASMVS exemplifies the idea *Civiltronics* [10], whereby traditional civil engineering concepts and knowledge is fused with key enabling technologies of the 4th Industrial Revolution to address challenging research problems.

**Fig. 1:**
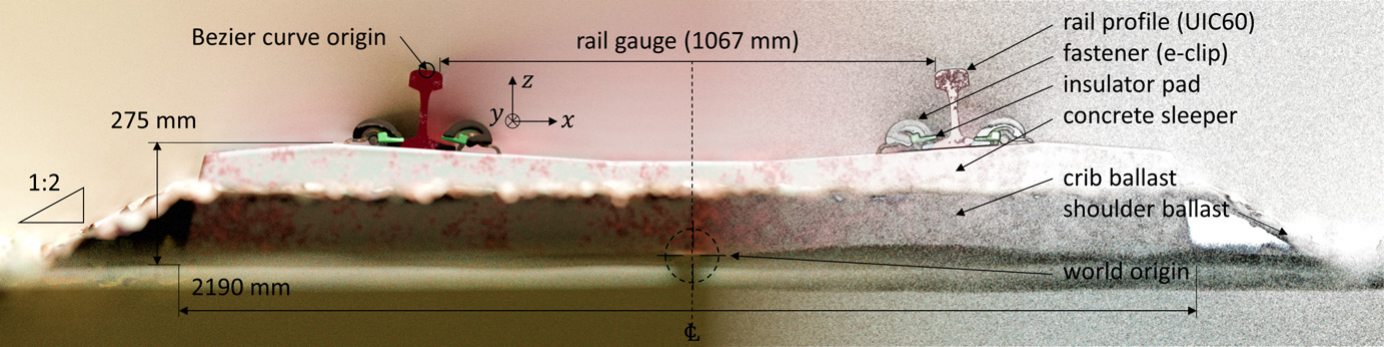
Cross sectional render illustrating the various superstructure components of the railway track, the ballast configuration and the world origin, along with the most relevant dimensions.

For the presented dataset, a 100 m length of tangent (linear) section of railway track is modeled (Fig. 1), comprising concrete sleepers (600 mm center-to-center spacing), e-clip fasteners, ground and ballast planes, insulator pads and rail profile (UIC60) with a gauge of 1067 mm (Cape gauge). The material shaders range from natural and diffuse for the ground, ballast and concrete, to a combination of rusted and specular metals for the rail profile and head of the rail, respectively. The vertical alignment (z-axis) of the two parallel rail profiles, along with the accompanying superstructure components, are defined by a Bezier curve with a point-to-point distance of 10 mm. The PSD sourced from representative track geometry data is used to generate random and unique vertical alignment data for the rail profile for each of the 40 scenes. This procedure is automated using the integrated Python API (application programming interface) within Blender.

**Fig. 2:**
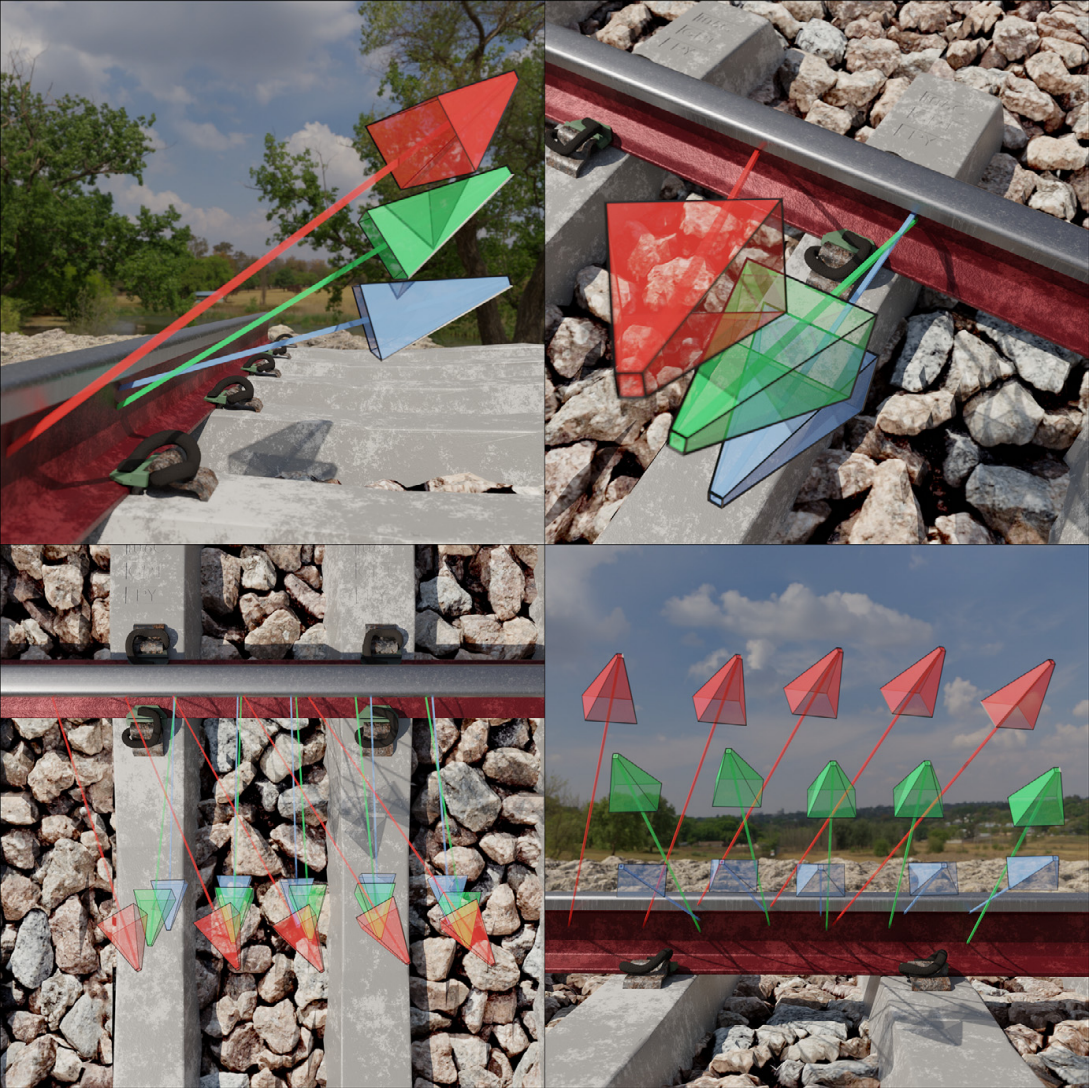
Illustration of the three primary camera positions and orientations observed from different perspectives. Projection of the center of the imaging sensor is represented by the ray emitted from every respective camera. (For interpretation of the references to color in this figure, the reader is referred to the web version of this article.)

Three camera positions were selected following extensive simulations that considered numerous possible camera configurations and their respective MVS accuracies (Fig. 2). The final configuration reflects the corresponding practical implementation of such a camera system on an instrumented vehicle. The x-coordinate (lateral) of the camera remains fixed along the center line of the track, with the z-coordinate (longitudinal) increasing in fixed increments of 148.5 mm along the length of the track. The bottom (blue), center (green) and top (red) cameras are positioned 100 mm, 250 mm and 400 mm above the railhead, respectively. The y-axis rotation (pitch) of the bottom, center and top cameras are 75, 60 and 55 degrees, respectively, centering the camera's imaging sensor and focus point about the web of the rail profile. The z-axis rotation (yaw) of the bottom and center cameras are perpendicular to the direction of the rail profile, whereas the top camera rotated by 30 degrees. This overlapping camera view configuration demonstrates a preference for a larger relative angle between the camera and rail to reduce the uncertainty volume, in addition to reducing the ambiguity along the edge of the reference image where views do not overlap.

**Fig. 3:**
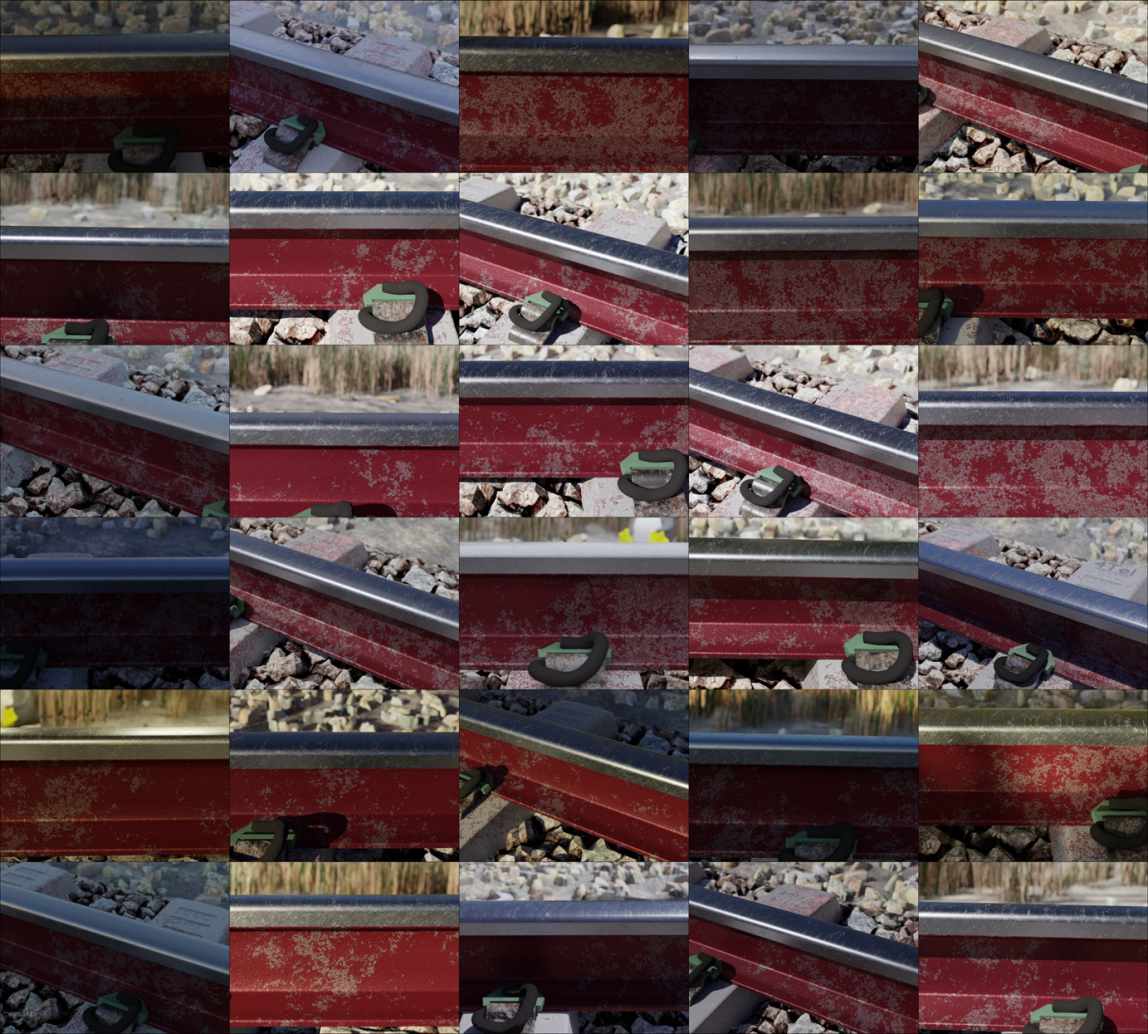
Random (stratified sampling) selection of renders, each from a different scene.

**Fig. 4:**
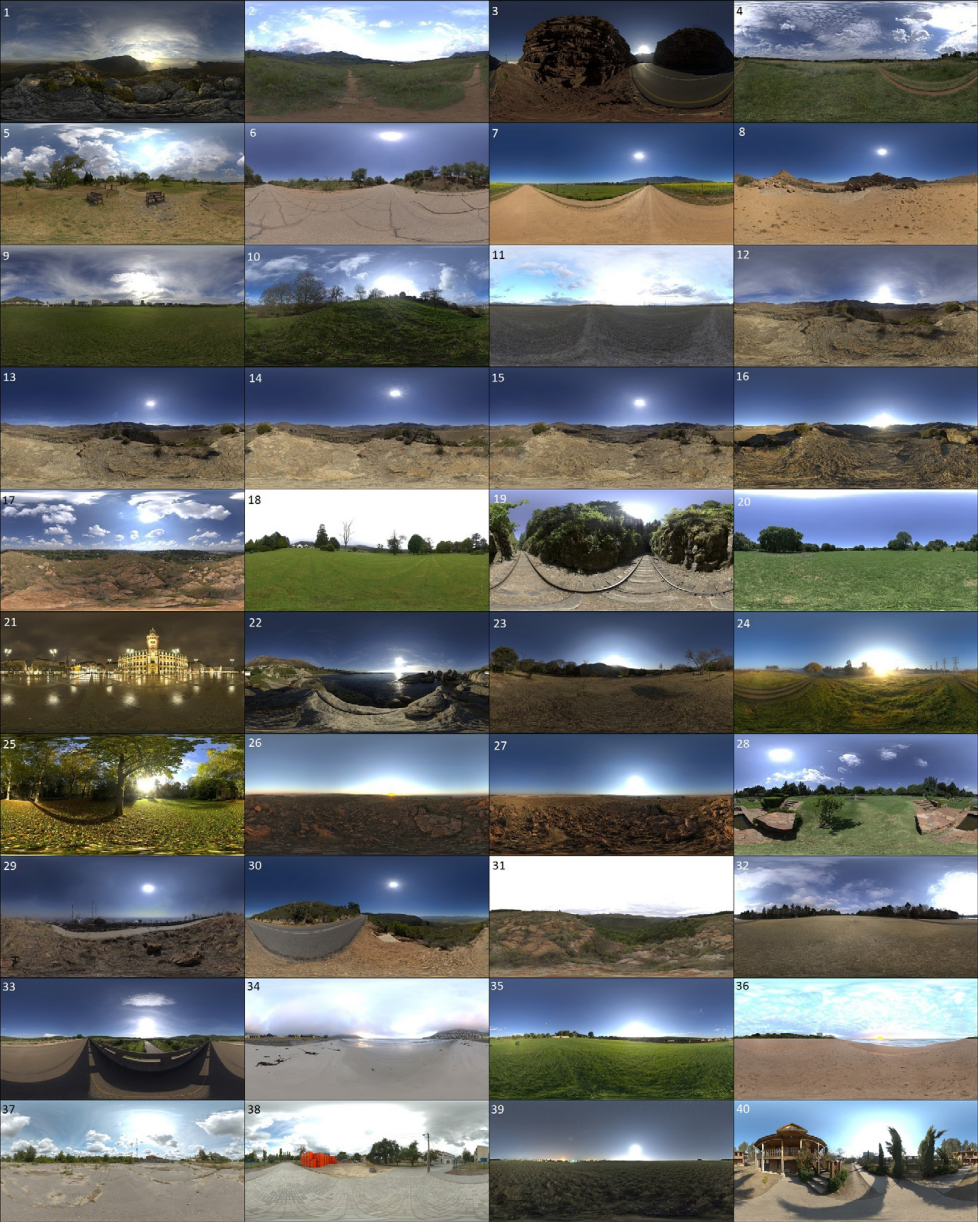
Illustration of the 40 high definition, HDR environmental lighting textures used for photorealistic scene illumination [13].

The focal length of the cameras varies between 58 and 61 mm (constant value used for all cameras for all frames of a particular scene), along with the material model of the railhead; minor variations are introduced for the steel shader. All of the superstructure components were modelled according to standardised dimensions used in industry. The fastener (e-clip) was digitised with a commercial VSLAM-based (Visual Simultaneous Localization and Mapping) desktop scanner [11,12]. Frame numbers for every scene range from 0 to 1994 (the bottom, center and top cameras start with frame numbers 0, 1 and 2 respectively), yielding a total of 79,800 colour renderings for the RailEnV-PASMVS dataset (Fig. 3). Every scene folder number, i.e. “19”, corresponds to the scene number (01 through 40). Forty, high-definition environmental textures (8K resolution) sourced from HDRI Haven [13] ensures accurate global illumination, replicating natural environments. Fig. 4 illustrates the equi-rectangular projections of the environmental textures along with their respective scene identification numbers.

The scenes for RailEnV-PASMVS are divided using a 80–20% train-validation split using a stratified sampling scheme. The list of all scenes, training scenes and validation scenes are stored in the *all_list.txt, training_list.txt* and *validation_list.txt* text files, respectively. The *index.csv* CSV file provides a convenient reference for all the sample files, linking the corresponding file and relative data path. The *RailEnV-PASMVS.blend* Blender source file, in addition to the 40 scenes (individual archives) are accessible from the data repository [2]. The camera information file for every scene is exported as a CSV file and stored in the scene folder as *scene.csv*. All signed float values are stored to a length of 5 decimal places. The following parameters are stored in the scene file:

• frame: frame number identification increasing from 0 through 1994 for every camera view.
• posX, posY, posZ: position vector (measured in meters) of the camera's origin point in Blender's world coordinate system; signed float.
• rotX, rotY, rotZ: rotation vector (measured in degrees, XYZ) of the camera coordinate system; signed float.
• resX, rexY: resolution (in pixels) of the sensor image; unsigned integer.
• focalLength: focal length of the camera (measure in millimetres); unsigned integer.
• sensorWidth: width of the camera's imaging sensor (in millimetres); unsigned integer.

For each unique scene folder, the output files are stored in four sub-folders, each detailed below. All image files (JPG file format) assume a fixed resolution of 768 × 576 pixels, with filenames padded to a fixed length of eight characters, e.g. *00001994.jpg*. The presented file format is identical to that detailed in PASMVS [1].

### 2.1 Blended_images

**Fig. 5:**
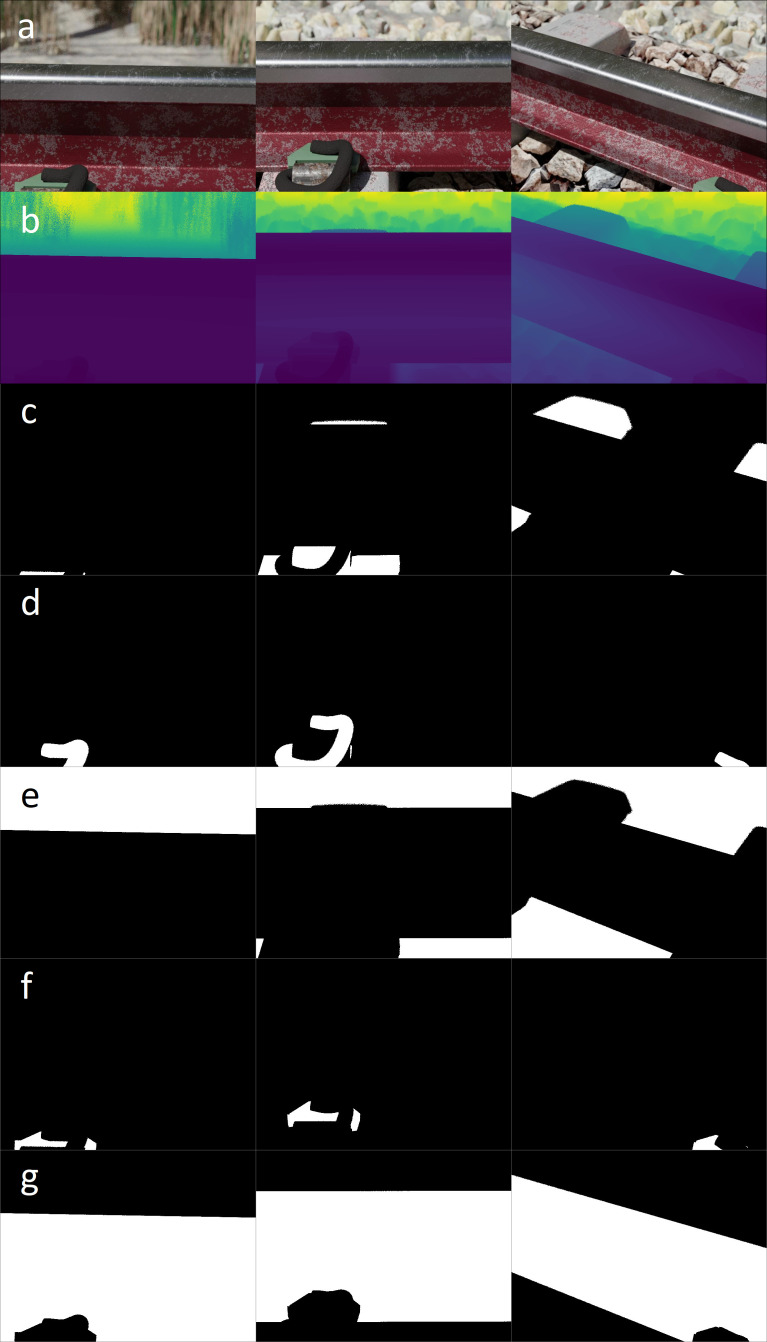
Illustration of the image rendering (row a), ground truth depth map (row b) and binary segmentation masks of the concrete sleeper (row c), e-clip fastener (row d), ground and ballast plane (row e), insulator pad (row f) and rail profile (row g) for all the primary camera perspectives.

Image renderings (e.g. *00001994.jpg*) of the railway environment rendered in perspective mode (Fig. 5a). The camera's sensor width is fixed at 36 mm with the focal distance varied between 58 and 61 mm.

### 2.2 Cams

The camera information, composed of a homogenous extrinsic and intrinsic matrix, is stored as a text file (e.g. *00001994.txt*) that corresponds to the image file name in *blended_images*. The last line of the camera text file specifies the minimum and maximum depth distance (first and last terms, respectively) alongside the step distance and number of depth hypotheses used for the neural network implementation [3] (second and third terms, respectively). The RailEnV-PASMVS data repository [2] contains the required information to transform the camera data from Blender's coordinate system into the correct matrices. An example of the camera file content is provided:

extrinsic
-0.0120252 0.8589704 0.0000507 -85.8280443
0.5047866 0.3009469 -0.8090868 -29.4175799
-0.6949969 -0.4142472 -0.5876891 41.8656260
0.0000000 0.0000000 0.0000000 1.0000000
intrinsic
1237.3333333 0.0000000 384.0000000
0.0000000 1237.3333333 288.0000000
0.000000 0.0000000 1.0000000
0.3319233 0.0055326 128.0 1.0401012

### 2.3 Masks

For every rendered image contained within the *blended_images* folder, a total of 5 corresponding binary segmentation masks are stored in the *masks* folder. The binary segmentation mask (comprised of black and white coloured pixels) encodes the occupancy of a particular pixel as viewed from the perspective of the camera. The masks for the concrete sleepers (e.g. *00001994cs.jpg*, Fig. 5c), e-clip fasteners (e.g. *00001994ec.jpg*, Fig. 5d), ground and ballast planes (e.g. *00001994gr.jpg*, Fig. 5e), insulator pads (e.g. *00001994ip.jpg*, Fig. 5f) and rail profile (e.g. *00001994cs.jpg*, Fig. 5g) are included.

### 2.4 Rendered_depth_maps

For every camera view stored in the *blended_images* folder, a corresponding ground truth depth map (e.g. *00001994.pfm*) is included (Fig. 5b) in the *rendered_depth_maps* folder. The rendered depth maps represent the distance measured from the camera's principal point to the intersecting scene geometry, for every pixel of the camera's imaging sensor [1,14].

### 2.5 Physical dataset

**Fig. 6:**
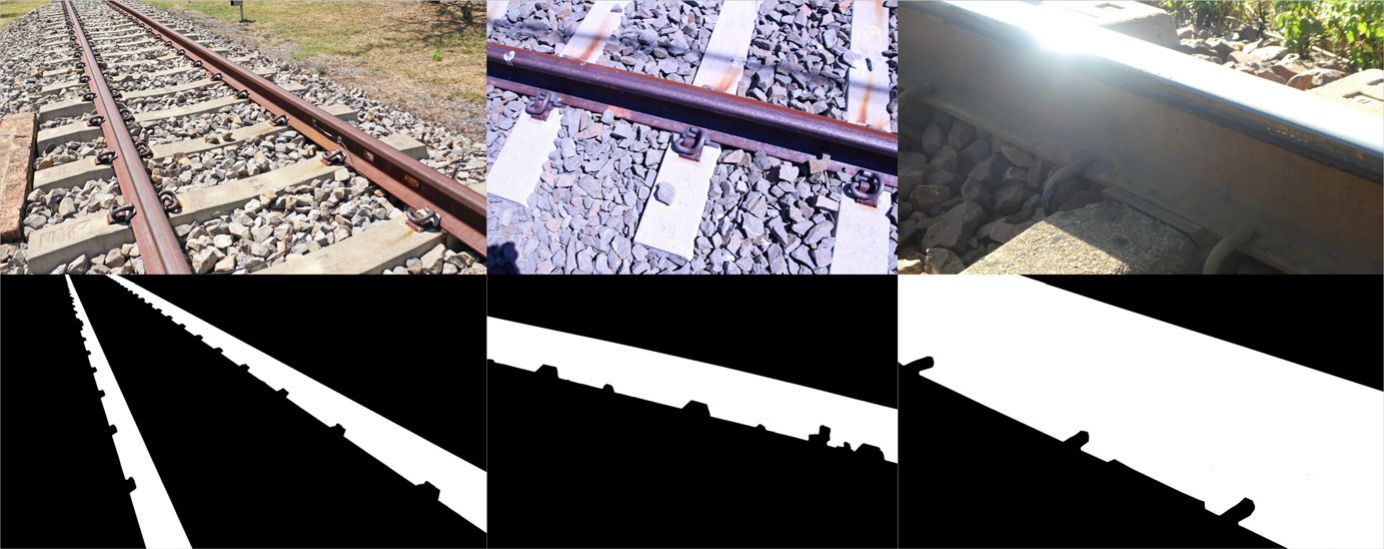
Illustration of samples (1st row) 10000049 (left), 10000099 (center) and 10000201 (right) for the physical dataset, alongside the corresponding manually annotated binary segmentation masks (2nd row).

The physical dataset is comprised of high-resolution photographs captured from both handheld digital cameras (Canon EOS 100D, 5184 × 3456 pixels; Panasonic DC-TZ90, 5184 × 2920 pixels), a UAV (DJI Mavic Air, 4056 × 2280 pixels) and computer vision cameras (Basler a2A1920-160uBAS, 1920 × 1200 pixels) from a variety of perspectives (Fig. 6, 1st row). Both traditional ballasted and PY slab track sections were considered with the railway components exhibiting varying degrees of rust for added variation and illumination conditions. The binary segmentation mask for each of the 320 full resolution photographs (Fig. 6, 2nd row) was manually annotated using Gimp, the open-source graphics editor, restricted to the rail profile owing to the time intensive nature of generating the data and limited use for other component classes. The physical dataset (*PhysicalDataset.zip*) is accessible from the data repository [2], with the photographs and masks subdivided into the *images* and *masks* folders respectively. Similar to the synthetic dataset, the filenames are padded to a fixed length of eight characters, e.g. *00000124.JPG* for both the colour photograph and corresponding binary segmentation mask. Unlike the synthetic dataset, the photographs and binary segmentation masks share the same filename, stored in the respective subfolders. The value of this complementary dataset is demonstrated in the last section (*Novelty*) of this article.

## 3 Experimental Design, Materials and Methods

### 3.1 Synthetic data

Blender is a free and open-source creation suite which serves as the primary end-to-end creative pipeline to create the RailEnV-PASMVS dataset. The availability of the path-traced rendering engine (Cycles) proved instrumental in realising the required photorealism and physically accurate rendering results required for diffuse and specular material shaders. The availability of high-resolution HDR environmental textures replaces the time-consuming process to develop realistic lighting conditions. The projection of the environmental textures remains static for every scene, avoiding any movement of shadows. Similar to the original PASMVS dataset, the configuration required for every scene (rebuilding the geometry of Bezier curve, replacement of background environmental textures, phase shifting noise modifiers and changing relative directories for output data) is accomplished with the Python API. The 148.5 mm interval between the successive camera positions along the length of track (with a sleeper spacing of 600 mm) provides an out-of-phase view sequence over the length of track, further increasing the data distribution. The f-stop (8) of the camera reflects the equivalent camera configuration expected to be implemented for experiment applications.

The pairing of camera views (*pair.txt* file located in the *cams* folder for every scene) follows a distinctive pattern for the three cameras (Fig. 2). This pattern remains viable for much of the track, except at the opposing ends where suitable perspectives are not available. The pair files accommodate these edge cases by duplicating the reference camera view. This approach, whilst providing poor inference during training for these extreme edge cases, seamlessly integrates with the current data structure requirements of MVSNet [3]. In practice these files are discarded, non-convergence of the SfM (structure from motion) optimisation algorithms.

Three levels of detail (LOD) were introduced for the virtual environment to optimize computational constraints:

• High LOD. High-density rail profile extruded in 10 mm segments, digitised e-clip fasteners and textured ballast materials with subdivision modifiers to realistically represent the geometry, without resorting to memory intensive particle emitters.
• Medium LOD. The immediate vicinity around the railway track includes individual ballast particles (20,000), grass (5000), large rocks (750) and sticks (500) to add detail, additional variability and feature markers visible from the perspective of the camera. The gras distributed along the sloped edges of the surrounding terrain serves as a visual backdrop with finite depth. For the depth map hypotheses (minimum and maximum depth), only the superstructure components are considered, confining the depth range hypotheses to the components of interest, disregarding the background environment as a result. For neural network training applications, this truncation serves as an effective means of regularization. The OHTE (overhead traction equipment) were modelled from simple geometric shapes and curve modifiers for realistic shadow casting onto the ground.
• Low LOD. Two planes were added to the opposing sides of the railway cutting with low resolution trees (200), adding the final layer of background detail.

### 3.2 Rendering

**Fig. 7:**
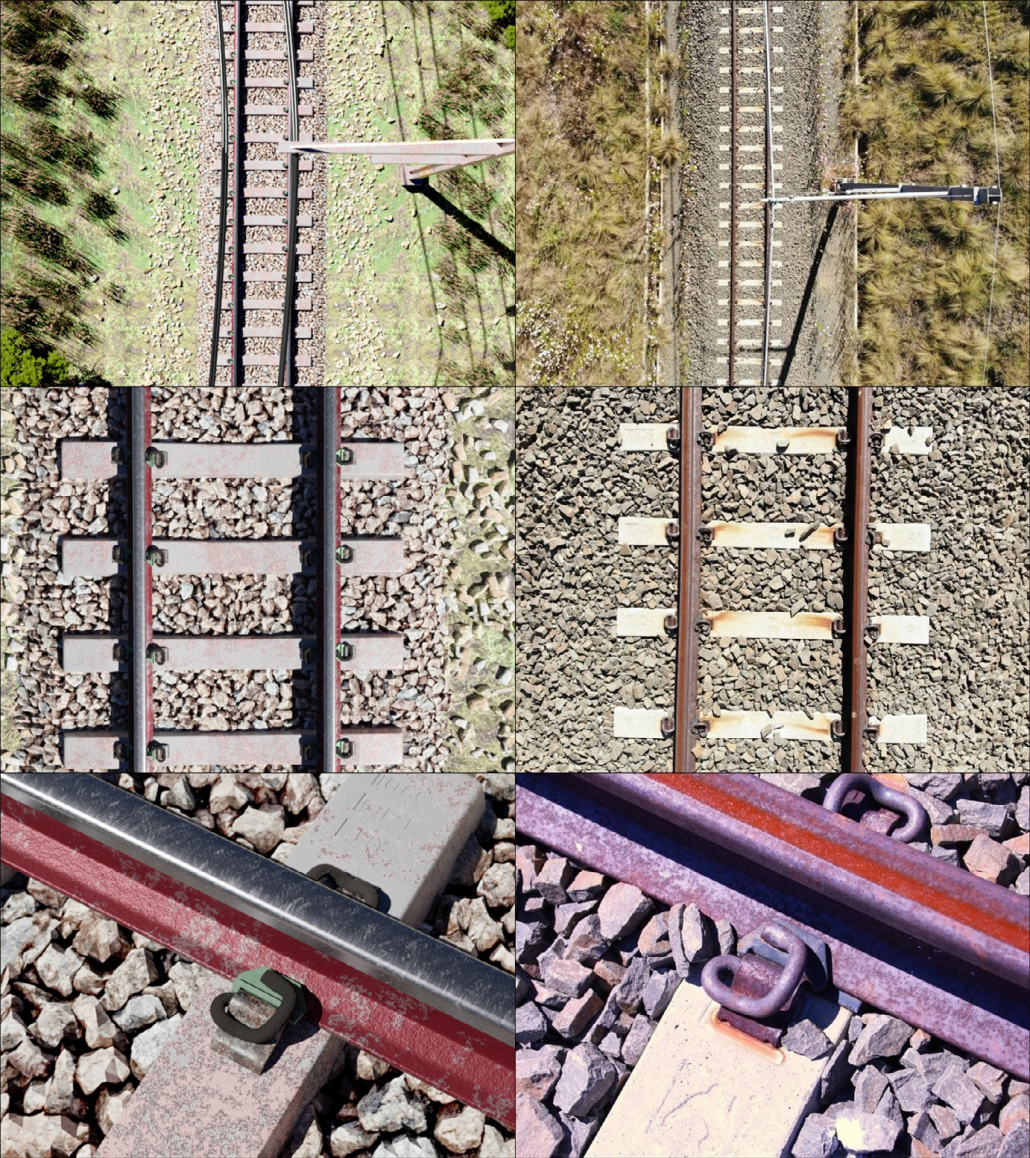
Comparison between the virtual environment created within Blender (left column) and photographs of railway components from similar perspectives (photo credit: Rick Vandoorne).

**Fig. 8:**
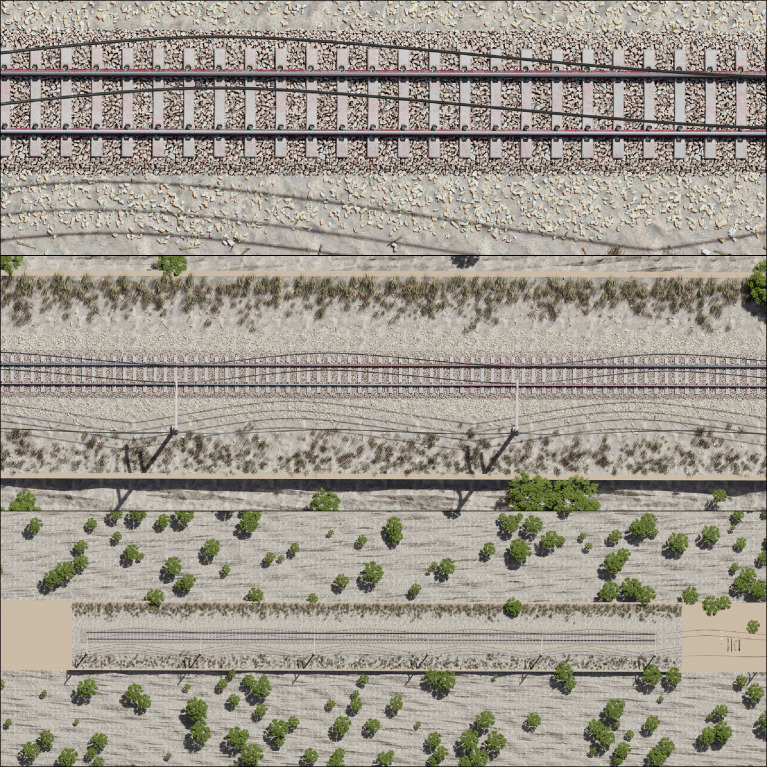
Nadir perspective of the entire virtual environment from an elevation of 25 m (top), 75 m (center) and 225 m (bottom).

PBR (physically-based rendering) materials, simplified geometry and low polygon-count assets were introduced as far as possible, improving the overall performance of the rendering process. Fig. 7 highlights the similarity between photographs acquired from a real railway track environment – which originally served as reference material during the design phase (Fig. 7, left-hand column) – and that of the virtual environment recreated with Blender (Fig. 7, right-hand column). Fig. 8 provides an overview of the entire virtual model, revealing the utility of dividing the environment according to different LODs to achieve acceptable rendering performance. The virtual environment is composed of 14.2 million vertices and 5.6 million faces, without consideration of the subdivision modifiers increasing the level of detail during rendering. Rendering of all 40 scenes spanned over a four-month period on a workstation-grade computer featuring an AMD Ryzen™ 9 3900 (12-core, 12-thread) CPU, Nvidia GTX1080 GPU and 48 GB of RAM.

### 3.3 Vertical geometry

**Fig. 9:**
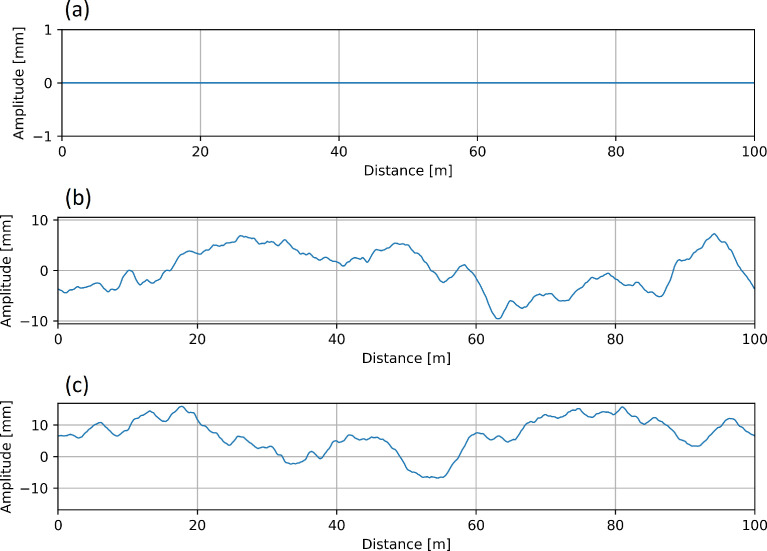
Synthetic profile geometry for (a) scene 1, (b) scene 19 and (c) scene 40 as a function of the track distance.

**Fig. 10:**
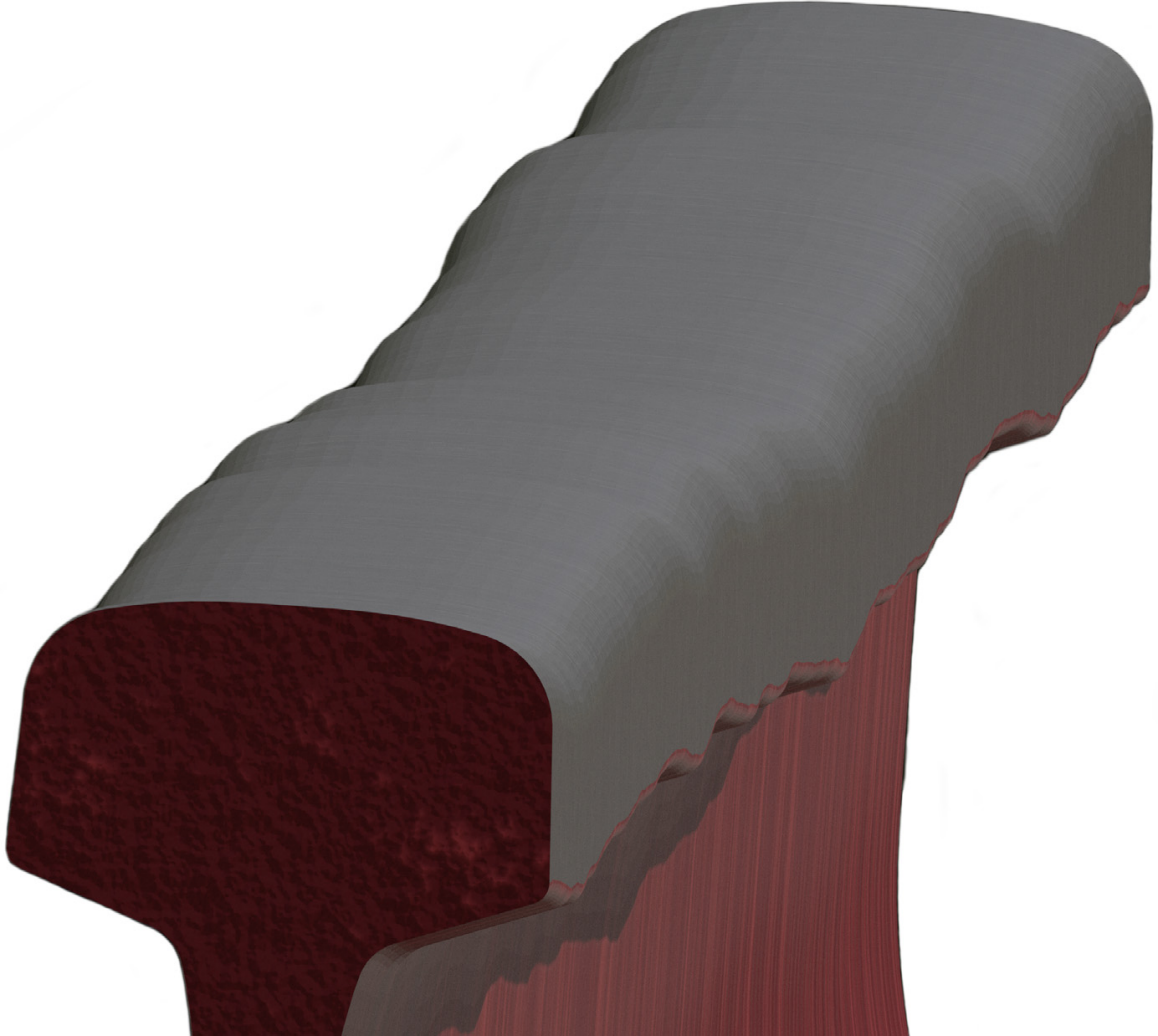
Orthographic perspective of the 100 m railway track.

The synthetic vertical geometry is applied to the railway track prior to rendering. The geometry data is stored in a CSV file (e.g. *vertGeometry_00000019.csv*) that specifies the longitudinal offset (distance along the track, in m) and vertical offset of the railhead (in mm). Fig. 9 illustrates select examples of the synthetic geometry for scene 00 (Fig. 9a) that functions as a control, scene 19 (Fig. 9b) and scene 40 (Fig. 9c). The orthographic projection of the rail profile (Fig. 10) reflects the deviations which correspond to the synthetic geometry data along the 100 m length of track.

### 3.4 Geolocation information

**Fig. 11:**
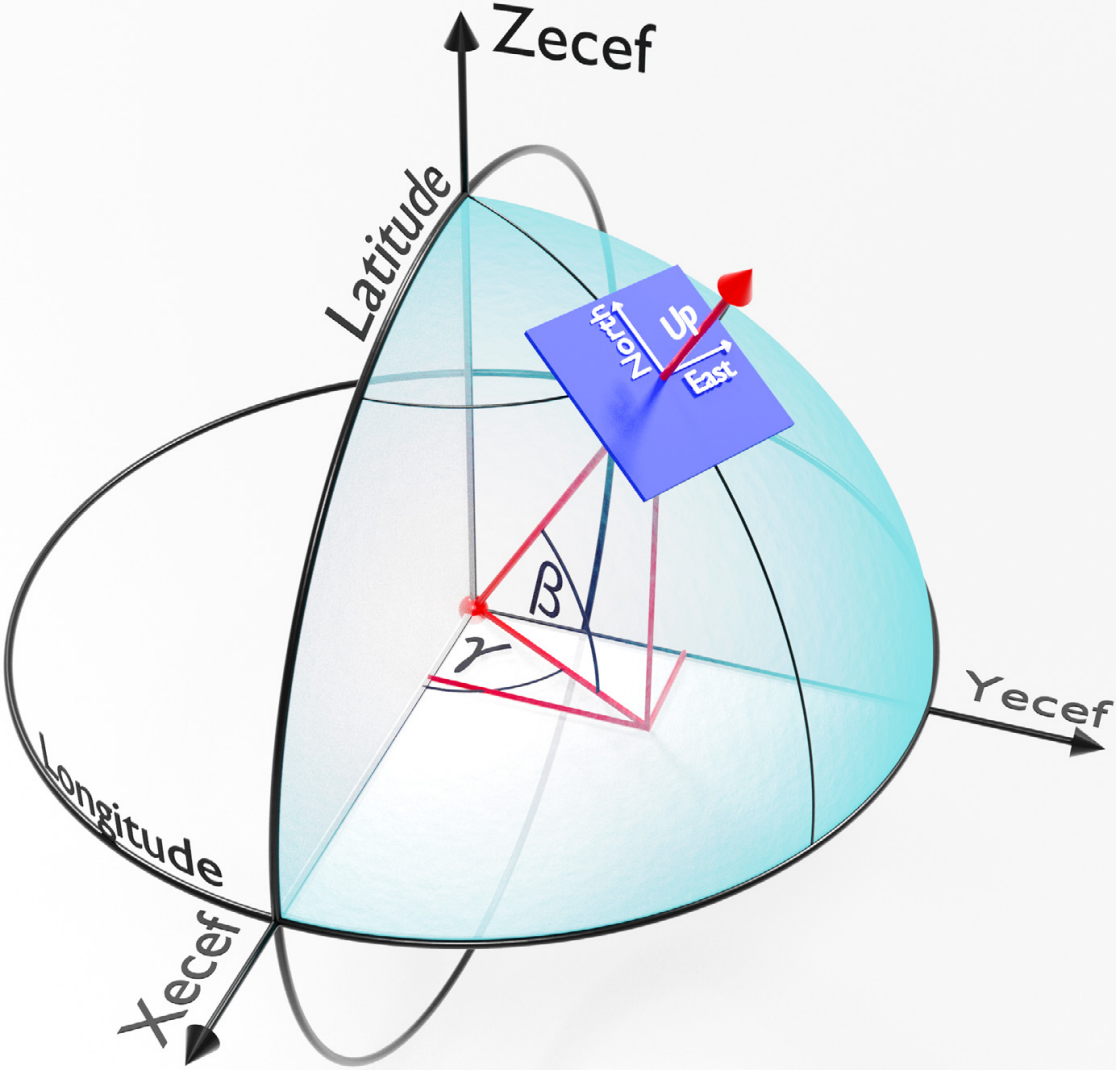
ECEF coordinate system in relation to latitude, longitude and the ECEF transformation of a local observer on the surface of the Earth.

**Fig. 12:**
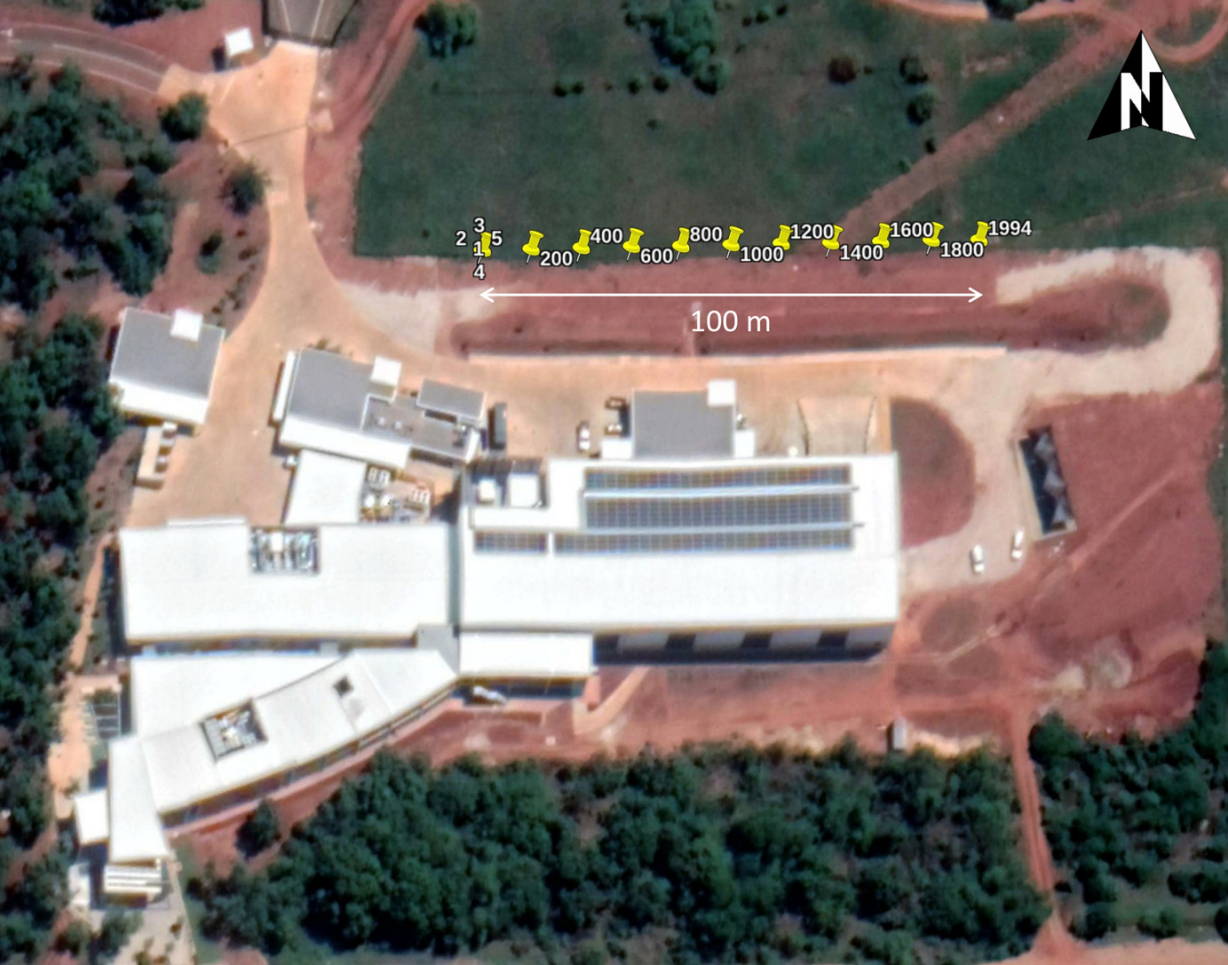
Mapped local coordinates to equivalent GPS coordinates adjacent to the Engineering 4.0 facility [17].

Earth-centered, earth-fixed (ECEF) is a Cartesian coordinate system that represents positional information using vectors (x-, y- and z-coordinates). Powerful photogrammetric software packages such as MicMac [15] benefit from additional geolocation metadata in the form of GPS (global positioning system) or LLH (latitude, longitude and height) coordinates [16], improving both the robustness of solvers and precision of photogrammetric reconstruction. The local coordinate system utilized by Blender's camera can be mapped to any arbitrary starting position on the Earth (GPS→ECEF). Vector addition of the stating position's equivalent ECEF coordinate and the local camera coordinate (transformed using applicable Euclidean rotations, refer to Fig. 11) provides the equivalent ECEF coordinate of the camera. This pseudo-geolocation (ECEF position transformed back to an equivalent GPS coordinate, ECEF→GPS) is in turn embedded within the EXIF of the rendered images. The geolocation metadata integrates seamlessly with photogrammetric pipelines, in contrast to PASMVS which does not embed any EXIF data. The starting position for the RailEnV-PASMVS dataset (LLH = [-25.74217, 28.25882, 1351.8] | ECEF = [5064706.0, 2722370.0, -2753948.0]) is located adjacent to the Engineering 4.0 complex [17] on the Hillcrest campus of the University of Pretoria in South Africa. Fig. 12 illustrates a selection of markers representing the view number of an arbitrary scene. The *pyproj* Python library automates this coordinate transformation (GPS→ECEF→GPS) and embedding of metadata within each respective image file. The local coordinate system from Blender was transformed such that the y-axis of the local tangent plane (LTP) is aligned with the line of longitude (Fig. 11), with increasing view numbers progressing from East to West (Fig. 12). The equivalent camera coordinates represented using the GPS coordinate system is provided separately in the *gps.csv* CSV file for each scene folder.

**Fig. 13:**
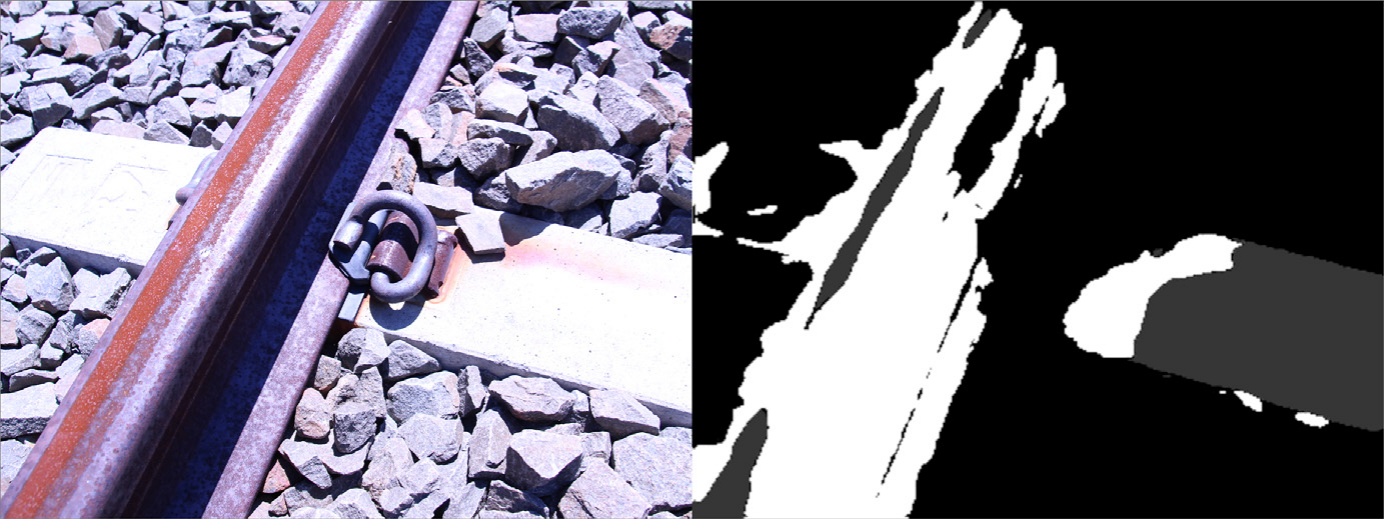
Validation sample (left) and segmentation inference (right) for multi-class segmentation.

**Fig. 14:**
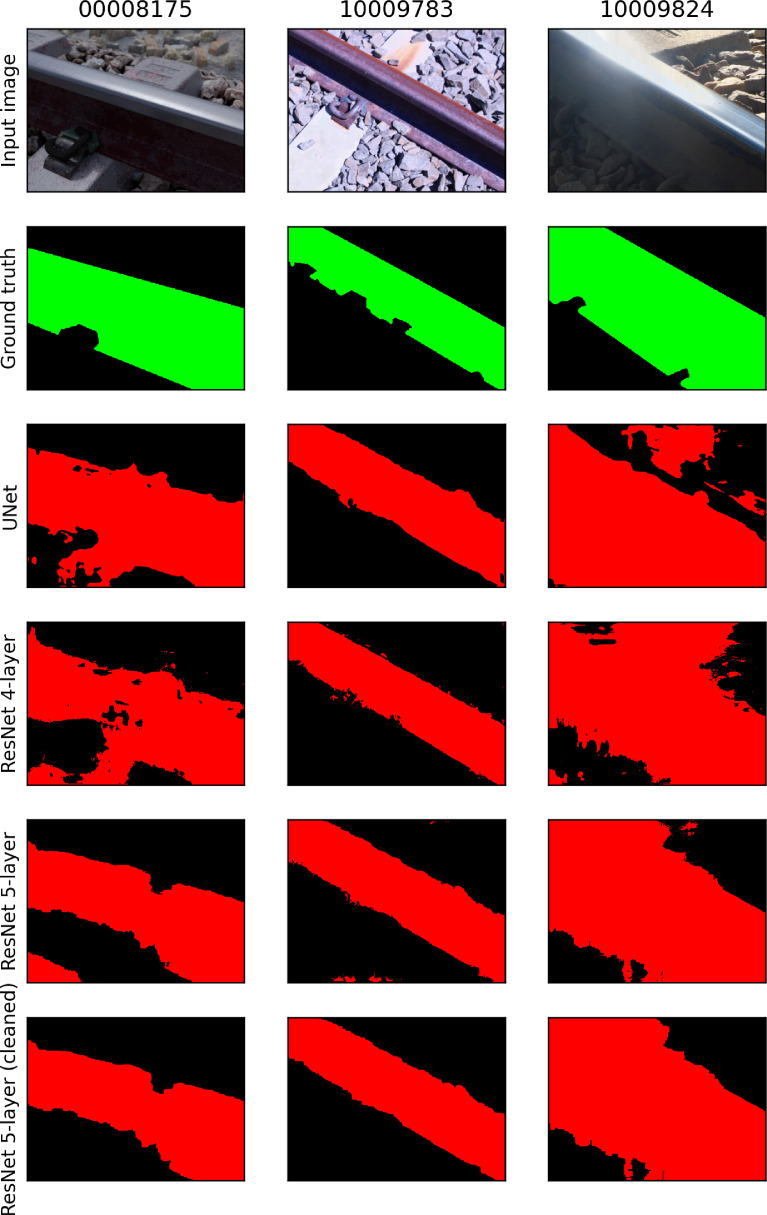
Validation images (1st row) and ground truth binary segmentation map (2nd row) compared to the inference results obtained from UNet (3rd row) and ResNet (4th, 5th and 6th row) architectures.

The equivalent geolocation functionality for practical scenarios will be provided by a Real-Time Kinematic (RTK) geolocation solution deployed close to the intended location. The incorporation of correction data from a fixed base station in close proximity to a GNSS receiver is a differential GNSS technique referred to as Real-Time Kinematic [18] navigation. The successful demonstration of a RTK geolocation service, that will be used for the explicit purpose of MVSNet reconstruction for railway infrastructure, has been successfully demonstrated [19].

### 3.5 Novelty

Two applications of the RailEnV-PASMVS dataset are demonstrated; the first demonstrates the benefits of combining synthetic and photographic data for semantic segmentation as part of a post-processing pipeline, with the second example illustrating the advantages when training the MVSNet neural network using domain specific data compared to other available datasets.

### 3.6 Semantic segmentation

Semantic segmentation automates the process of isolating features of interest; for railway applications, the component of interest is primarily the rail profile. This masking operation, automated using a trained neural network, can remove irrelevant points from the corresponding depth map inferred by MVSNet for such an application. The generalisation performance of a neural network trained exclusively on synthetic data for all 5 component classes (rail profile, e-clip fastener, insulator pad concrete sleeper, ballast and ground surface) proved insufficient when tested on photographs of railway components (Fig. 13).

An alternative approach was explored to sample half of the training examples from the synthetic dataset and the other half from the photographs, using only one component class (rail profile) instead of all five. Using Python, a total of 10,000 images (768 × 576 pixels) were sampled from the 320 high-resolution photographs; these high-resolution images were scaled between 30% and 80% of their original size, followed by a random cropping operation of the desired resolution (for both the photograph and the corresponding hand-annotated binary segmentation mask). The samples were divided using a 80–20% train-validation split using a random sampling scheme.

Fig. 14 illustrates the improved performance using the mixed dataset. The input images (Fig. 14, 1st row) are representative of both the synthetic dataset and rusted (diffuse) / reflective (specular) rail photographs. The corresponding hand-annotated ground truth binary segmentation maps are also illustrated for reference (Fig. 14, 2nd row). Three variations (UNet, ResNet 4-layer and ResNet 5-layer) of the popular UNet [20] neural network architecture was evaluated. The implementation producing the best qualitative inference (ResNet 5-layer) underwent a final post-processing step (using OpenCV) to remove noise and small artifacts (Fig. 14, 6th row). The advantage of increasing the variation of the training data through combining synthetic data and real photographs is evident.

**Fig. 15:**
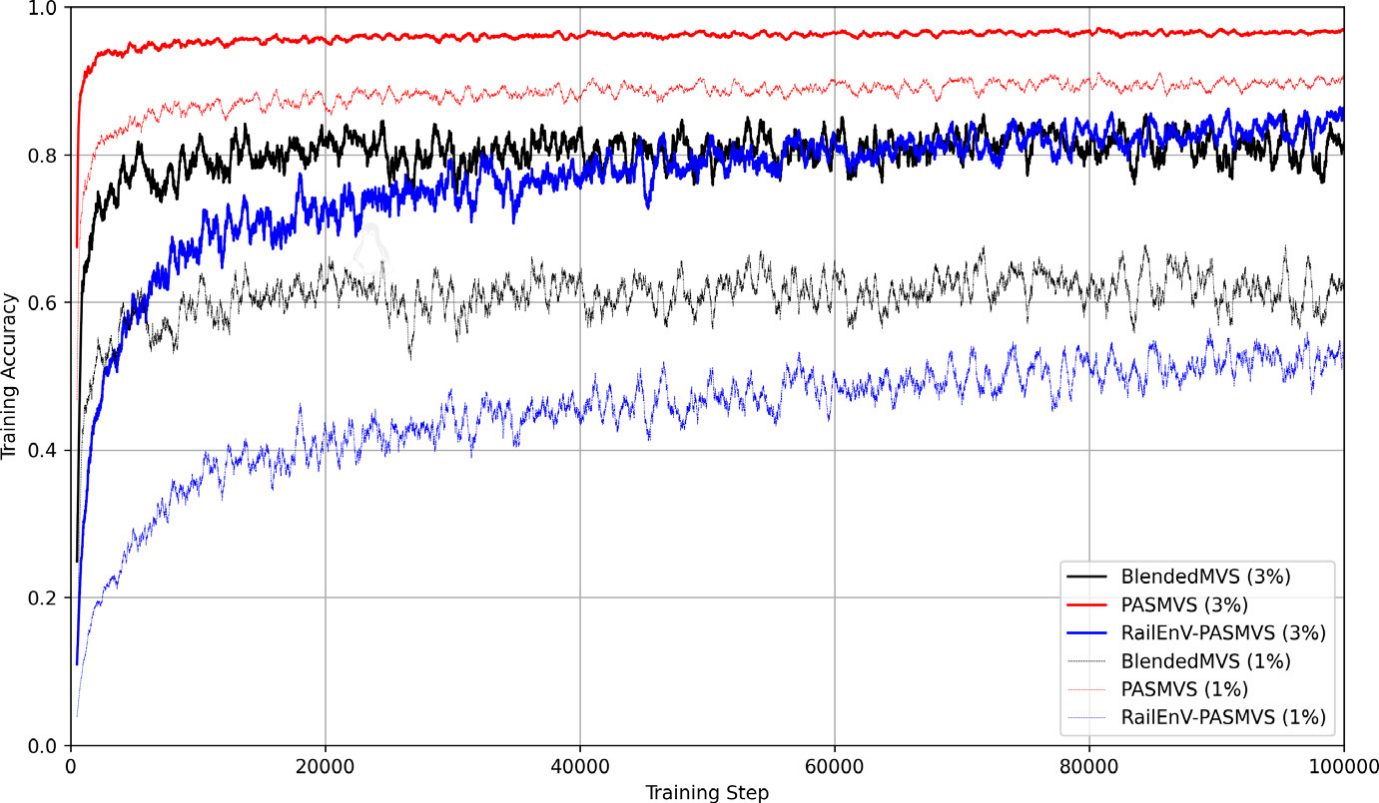
Training statistics associated with MVSNet trained with BlendedMVS, PASMVS and RailEnV-PASMVS datasets.

**Fig. 16:**
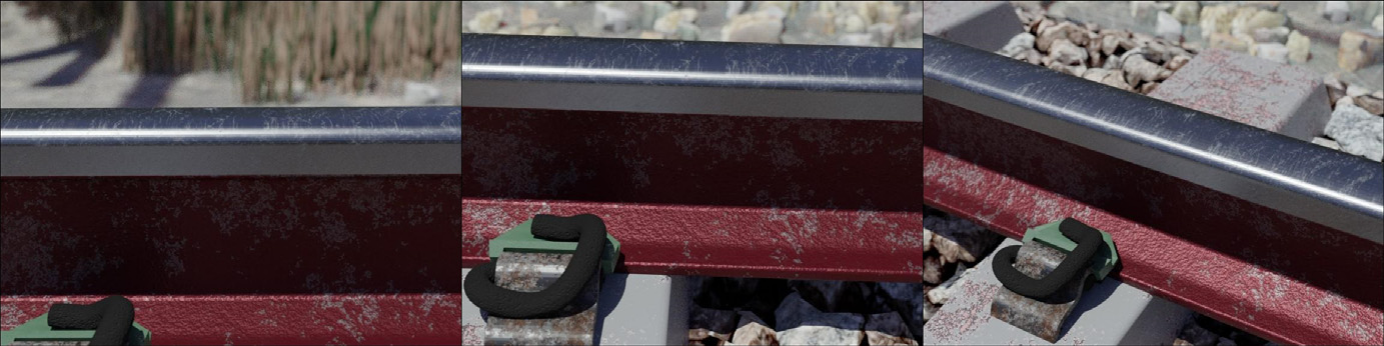
Test set for the pointcloud reconstruction sourced from RailEnV-PASMVS scene 06: reference view 13 (center), 12 (left) and 20 (right).

### 3.7 MVSNet inference performance

Comparing the training history (Fig. 15) of MVSNet for each of the three datasets discussed (BlendedMVS, PASMVS and RailEnV-PASMVS) illustrates the varying complexity of each dataset. The hyperparameters were kept fixed for all three training scenarios (3 views, 128 depth hypotheses). PASMVS attains the highest training accuracy, followed by BlendedMVS with RailEnV-PASMVS trailing in third over the course of 100,000 training iterations.

**Fig. 17:**
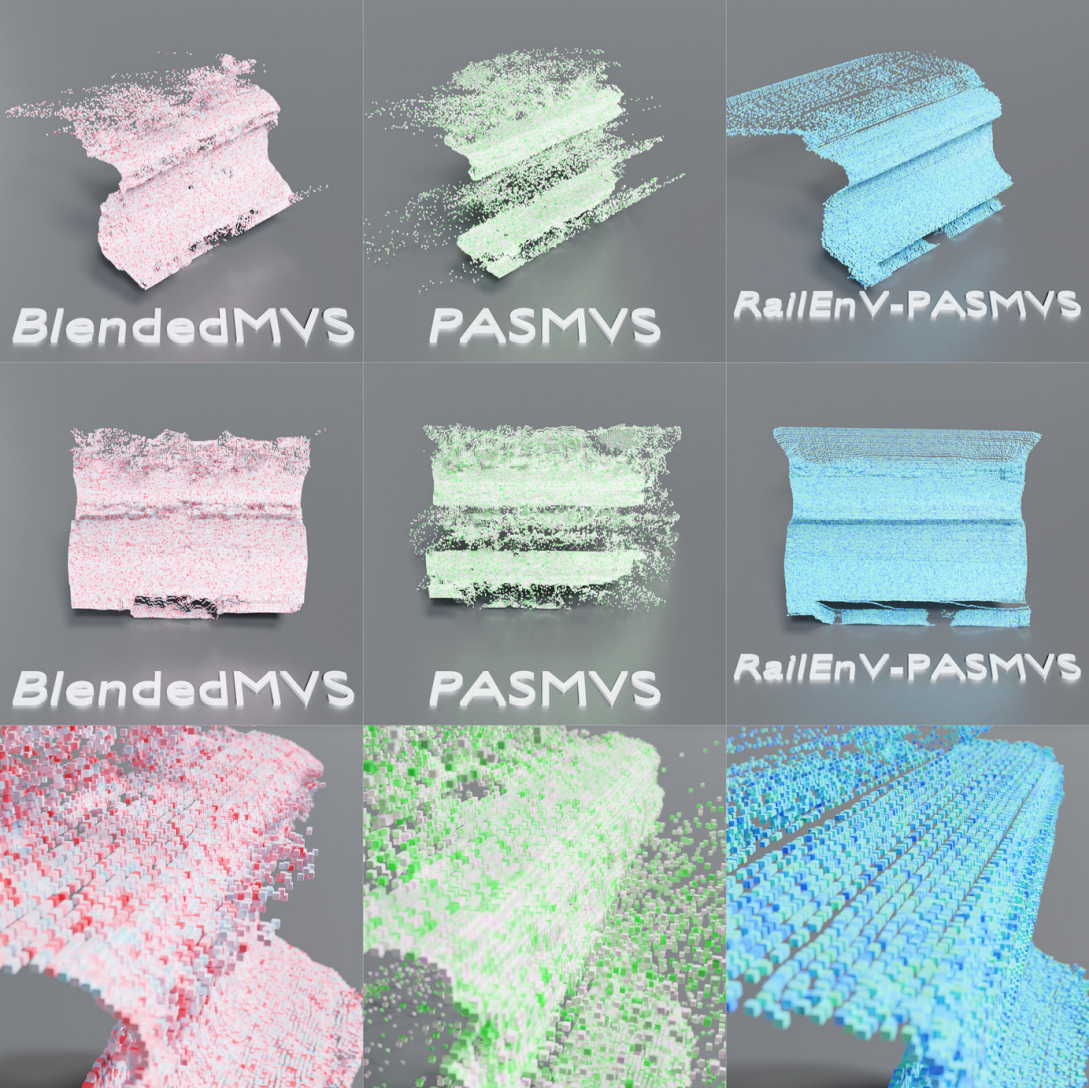
Qualitative reconstruction quality comparison for the MVSNet neural network trained on the BlendedMVS (left column), PASMVS (center column) and RailEnV-PASMVS (right column).

An example of the reconstructed point clouds for one of the validation scenes from RailEnV-PASMVS (Fig. 16), each generated by the three respective networks, is illustrated (Fig. 17) from three different perspectives. The point clouds are the corresponding projected inference depth map in three-dimensional space, with each vertex represented by a small cube for improved visualisation. BlendedMVS (Fig. 17, left-hand column) provides a comparatively good quality reconstruction except for the specular railhead yielding noisy depth proposals. PASMVS (Fig. 17, center column) performs poorly for the shadowed regions, a feature that is noticeably absent from corresponding dataset, in addition to an undesirable wavelike discontinuity associated with the railhead. RailEnV-PASMVS (Fig. 17, right-hand column) resolves these shortcomings, producing a much more accurate representation of the rail profile, in particular for the more challenging specular features. However, it should be recognised that this validation sample is derived from the same underlying data distribution (of the rail environment). Nonetheless, with the expected distribution of training data known a-priori, the quality of the reconstructions should prove to be equally favourable for data sourced from photographs sourced from field experiments. Research efforts are currently directed toward evaluating the performance of the neural networks trained on the synthetic datasets when presented with equivalent experimental data obtained from real railway environments.

## CRediT authorship contribution statement

**André Broekman:** Conceptualization, Data curation, Formal analysis, Investigation, Methodology, Software, Validation, Visualization, Writing – original draft, Writing – review & editing. **Petrus Johannes Gräbe:** Funding acquisition, Project administration, Resources, Supervision, Writing – review & editing.

## Declaration of Competing Interest

The authors declare that they have no known competing financial interests or personal relationships which have, or could be perceived to have, influenced the work reported in this article.
